# Myositis Ossificans Circumscripta Without History of Trauma

**DOI:** 10.4021/jocmr2010.05.364w

**Published:** 2010-06-15

**Authors:** Jose Aneiros-Fernandez, Mercedes Caba-Molina, Salvador Arias-Santiago, Francisco O'Valle, Pedro Hernandez-Cortes, Jose Aneiros-Cachaza

**Affiliations:** a Department of Pathology, University Hospital, Granada, Spain; b Department of Dermatology, University Hospital, Granada, Spain; c Department of Orthopaedic, University Hospital, Granada, Spain

## Abstract

Myositis ossificans circumscripta is a form of heterotopic ossification that is benign in nature associated to a trauma, but may appear clinically and radiologically as a malignant neoplasm. We describe a rare case of calcifying of myositis ossificans not associated to trauma in a 35-year-old woman with a mass in her upper third and external of right thigh. We discuss some of the difficulties of diagnosis and histological evolution of the lesion.

**Keywords:**

Myositis ossificans; Thigh; Differential diagnosis; Nontraumatic

## Introduction

Myositis ossificans circumscripta (MOC) is a form of heterotopic ossification that is benign in nature but may appear clinically and radiologically as a malignant neoplasm [1]. It is most frequently encountered in the arm, shoulder, thigh, and hand, in order of frequency. MOC may be linked to certain hematologic abnormalities as there is a report of heterotrophic bone formation developed as a long-term complication of iliopsoas hemorrhage in patients with hemophilia [2, 3]. Radiologically, it is difficult to distinguish this condition from soft tissue and bone malignancy, so a biopsy is necessary to confirm the diagnosis. We present a case of myositis ossificans occurring in upper third and external of right thigh without a history of trauma and no family history of interest, without cortical involvement, and suspected of being malignant soft tissues.

## Case Report

**Figure 1.**
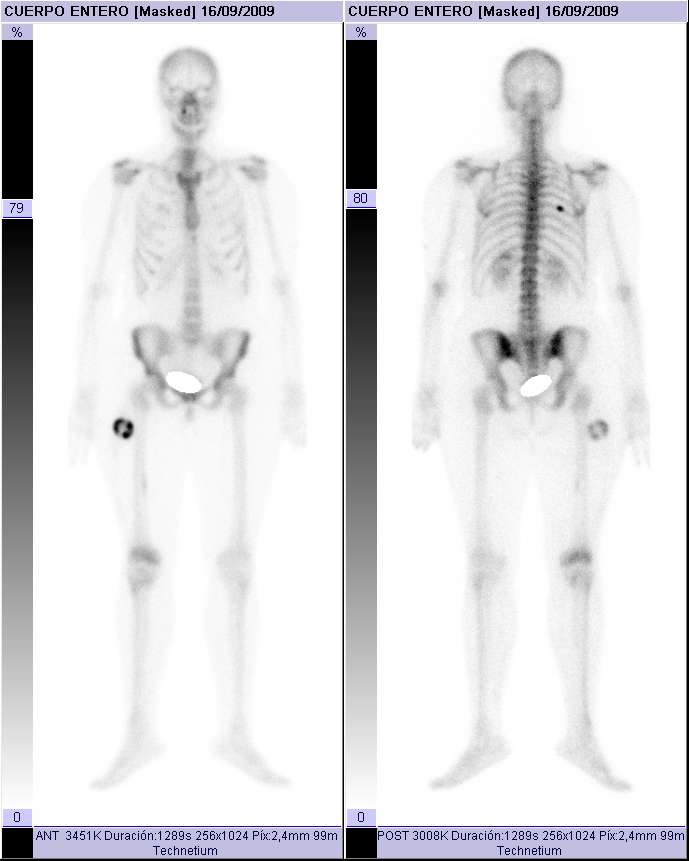
Bone gammagraphy presented intense uptake in upper third and external of right thigh.

**Figure 2.**
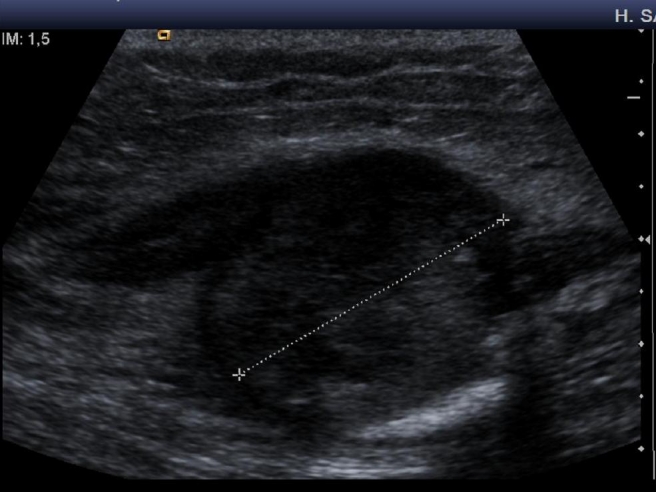
Echography revealed heterogeneous echogenicity with some calcification within it.

**Figure 3.**
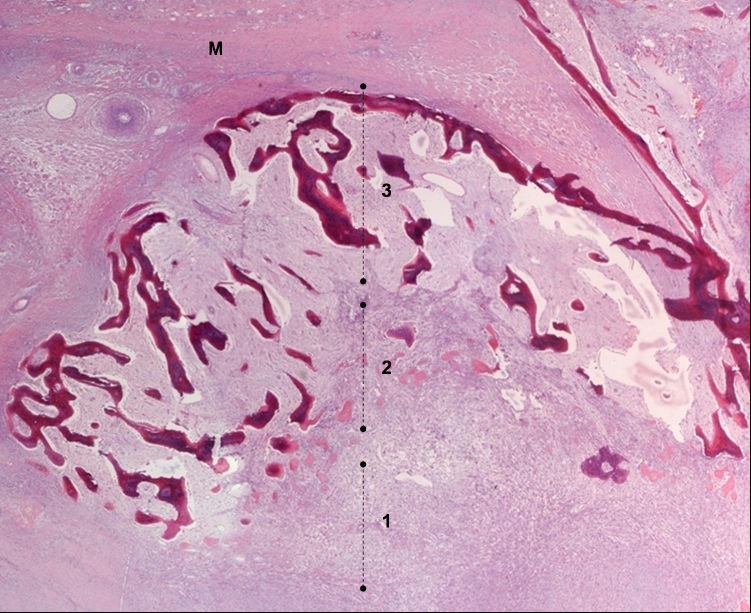
Showed three typical zones proliferating fibroblasts: (1) immature osteoid; (2) and bone formation at the peripheral; (3) Muscle tissue (M) (Panoramic, Hematoxiline and Eosine).

A 35-year-old caucasic woman, no previous personal or family history of interest, visited our hospital and presented upper third and external of right thigh mass. On physical examination, a slightly tender, hard, not well-circunscribed mass was palpated in the external of right thigh. She denied any previous history trauma including exercise-related trauma. The laboratory findings were normal. Computed tomography of the thigh showed a 42 × 27 mm mass. A faint calcification was noted in the peripheral area of the mass. Bone gammagraphy presented intense uptake in soft tissue with central area low uptake (Fig. 1). Echography revealed heterogeneous echogenicity with some calcification within it (Fig. 2). Histological showed three zones: (1) centre zone presented proliferating fibroblasts with areas of haemorrhage; (2) intermediate zone was characterized by osteoblasts with immature osteoid formation and islands of cartilage; (3) peripheral zone was composed of mature bone surrounding tissue by fibrous and muscle tissue (Fig. 3, 44).

**Figure 4.**
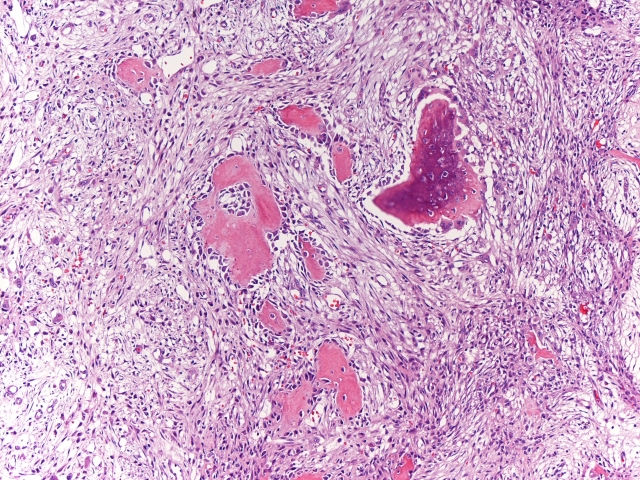
Osteoblasts with immature osteoid formation and islands of cartilage (magnification x 20, Hematoxiline and Eosine).

## Discussion

Nontraumatic MOC is a rarely reported benign heterotopic ossification characterized by the aberrant formation of bone in extraskeletal soft tissues. It is usually confined to a single muscle or muscle group, and is most common in active male within the second and third decade of life [4]. It usually occurs after muscle injury such as repeated microtrauma, but it can also occur without previous trauma. In a small number of cases, possible etiologies include infections, burns, neuromuscular disorders, hemophilia (factor-IX deficiency), tetanus, and drug abuse [5-7].

MOC is essentially a proliferative mesenchymal response to an initiating injury to the soft tissue, not necessarily to the muscle, which leads to localized ossification. In the first week, richly vascularised, proliferative fibroblastic cells are prominent. These primitive mesenchymal cells, with high mitotic activity, can mimic malignancy on biopsy. With maturation of the lesion, which is variable, a typical zonal pattern develops with three distinct zones: (1) the centre consists of rapidly proliferating fibroblasts with areas of haemorrhage and necrotic muscles; (2) the intermediate or middle zone is characterized by osteoblasts with immature osteoid formation and islands of cartilage due to enchondral ossification; (3) the peripheral zone is composed of mature bone, usually well separated from the surrounding tissue by myxoid fibrous tissue. Then, by the third to fourth week, calcifications and ossifications appear inside the mass; by the sixth to eighth week, a well-organized cortical bone with cortex and marrow space formation develops at the periphery [8-11].

Computed tomography is the gold standard in characterizing typical findings of myositis ossificans such as extensive muscle and perilesional edema without bone marrow or cortical abnormalities. The magnetic resonance imaging (MRI) findings vary dependent on the stage of maturation and the histologic pattern of the lesion. For early stage lesions, T2-weighted images show a heterogeneous localized mass with high signal intensity in the central area. As the lesion matures, the peripheral ossification becomes denser, and the T2-weighted MRI shows a hyperintense area with surrounding hypointense rim. On T1-weighted images, the lesion is isointense to the muscle and can be identified by mass effect but it may demonstrate rim enhancement in the acute phase after contrast administration [5].

Histologically, the differential diagnosis should include osseous osteosarcoma and fibro-osseous pseudotumour of digits. MOC tends to show a typical zonal pattern with a fibroblastic center and a broad zone of ossification at the periphery, while the central portion tends to show loosely arranged spindle cells with no cytologic atypia. Osteosarcomas have a disorderly growth with a reverse zoning effect. Immature woven bone or osteoid is found on the periphery, and mature osseous trabeculae are centrally located and also show proliferating cells with cytological malignant features.

The most common treatment of MOC is a surgical excision. Various treatments such as physical therapy, acetic acid iontophoresis treatment, magnesium therapy, and etidronate disodium have been reported to be effective [12-14].

Nontraumatic MOC of the upper third and external of right thigh is rare, and a thorough knowledge of the clinical-morfological study is necessary to differentiate this lesion from a malignant soft tissue tumor.
